# *fagin*: synteny-based phylostratigraphy and finer classification of young genes

**DOI:** 10.1186/s12859-019-3023-y

**Published:** 2019-08-27

**Authors:** Zebulun Arendsee, Jing Li, Urminder Singh, Priyanka Bhandary, Arun Seetharam, Eve Syrkin Wurtele

**Affiliations:** 1 Department of Genetics Development and Cell Biology, Iowa State University, Ames, IA 50010 USA; 2 Center for Metabolic Biology, Iowa State University, Ames, IA 50011 USA; 3 Genome Informatics Facility, Office of Biotechnology, Iowa State University, Ames, IA 50011 USA; 4 Bioinformatics and Computational Biology Program, Iowa State University, Ames, IA 50011 USA

**Keywords:** Synteny, Orphan, de novo, Software, Genome, And RNA-Seq

## Abstract

**Background:**

With every new genome that is sequenced, thousands of species-specific genes (orphans) are found, some originating from ultra-rapid mutations of existing genes, many others originating de novo from non-genic regions of the genome. If some of these genes survive across speciations, then extant organisms will contain a patchwork of genes whose ancestors first appeared at different times. Standard phylostratigraphy, the technique of partitioning genes by their age, is based solely on protein similarity algorithms. However, this approach relies on negative evidence ─ a failure to detect a homolog of a query gene. An alternative approach is to limit the search for homologs to syntenic regions. Then, genes can be positively identified as de novo orphans by tracing them to non-coding sequences in related species.

**Results:**

We have developed a synteny-based pipeline in the R framework. *Fagin* determines the genomic context of each query gene in a focal species compared to homologous sequence in target species. We tested the *fagin* pipeline on two focal species, *Arabidopsis thaliana* (plus four target species in Brassicaseae) and *Saccharomyces cerevisiae* (plus six target species in Saccharomyces). Using microsynteny maps, *fagin* classified the homology relationship of each query gene against each target genome into three main classes, and further subclasses: *AAic* (has a coding syntenic homolog), *NTic* (has a non-coding syntenic homolog), and *Unknown* (has no detected syntenic homolog). *fagin* inferred over half the “*Unknown*” *A. thaliana* query genes, and about 20% for *S. cerevisiae*, as lacking a syntenic homolog because of local indels or scrambled synteny.

**Conclusions:**

*fagin* augments standard phylostratigraphy, and extends synteny-based phylostratigraphy with an automated, customizable, and detailed contextual analysis. By comparing synteny-based phylostrata to standard phylostrata, *fagin* systematically identifies those orphans and lineage-specific genes that are well-supported to have originated de novo. Analyzing within-species genomes should distinguish orphan genes that may have originated through rapid divergence from de novo orphans. *Fagin* also delineates whether a gene has no syntenic homolog because of technical or biological reasons. These analyses indicate that some orphans may be associated with regions of high genomic perturbation.

**Electronic supplementary material:**

The online version of this article (10.1186/s12859-019-3023-y) contains supplementary material, which is available to authorized users.

## Background

One of the surprises of the genomic era was that gene birth is not a dead process. The prior paradigm, that proteins evolve only by gradual “tinkering” with existing material [1], was contradicted when the sequencing of the first genomes uncovered many species-specific “orphan” genes [2]. Most researchers argued then that the uniqueness of these genes was an artifact of sparse sampling or bad gene prediction, and that when enough genomes were sequenced, all correctly annotated genes would cluster into large, ancient families. But more sequencing proved exactly the opposite. Researchers have shown that not only can genes encoding novel proteins arise de novo [2, 3], but they do so often, as shown, for example, in animals [4–6], plants [7], protists [8], and yeast [9]. In addition to arising de novo, orphan genes could be derived from a very rapid mutation of existing CDSs beyond recognition [10], although we are unaware of specific evidence for this phenomenon.

Although most of the approximately several billion orphan genes in extent eukaryotes [11] have never been studied, functions are being shown for a growing minority. The emerging theory is that young genes are common in arenas where fitness optima change quickly, such as environmental response and inter-species relations. Orphans are over-represented among genes that respond to stress [12–15]. They may also be major contributors to taxonomically-restricted traits [16, 17]. Orphans also may play important roles in developmental cascades [18]. Other orphans are crucial to interspecies conflicts [19], self-incompatibility [20], host-pathogen relations [21], and symbiosis [22, 23]. One of the best-studied orphan genes, QQS of *Arabidopsis thaliana*, responds to biotic stresses by altering carbon and nitrogen partitioning [15, 24] and by conferring broad-spectrum pest and pathogen resistance [12]. A study of three de novo genes in mice, randomly selected from among very young genes that were inferred to be of de novo origin, found evidence of associated phenotypes (longer limbs, changed behavior, and slower life history) [25].

In addition to studies revealing the function of individual orphan genes, there is experimental evidence that functional, beneficial proteins can be produced from random sequence.

First, in vitro protein evolution from random protein libraries demonstrates that functional proteins can be produced through chance mechanisms [26–29]. Second, expression of randomly-generated ORFs in vivo can lead to phenotypic consequences. About 50% of random ORFs expressed in *E. coli* inhibited growth rate, while about 25% increased growth rate [30]. Of 2000 *A. thaliana* plants expressing random ORFs, ten biologically-relevant phenotypes were revealed and experimentally verified, including early flowering and red light insensitivity [31].

If new genes can arise de novo, new genes are constantly appearing, then some should survive across speciation events. Thus, genes in extant species should be stratified into sets of genes that appeared at different times. The technique of inferring the evolutionary time of origin of each gene across a genome is known as phylostratigraphy [32]. Phylostratigraphy is the study of the distribution of gene birth events across deep time by stratifying modern genes by age. In standard phylostratigraphy, the phylostratum of a given protein-coding gene is based on the age of the oldest clade that contains its inferred protein-coding homolog (e.g., [33]). Phylostratigraphy has been used to link clusters of clade-specific genes to the origins of clade-specific traits, such as brain development [33] or the early origins of cancer genes [34]. It also offers snapshots of proteins of different ages and thus provides a unique window into protein evolution, offering insight into the evolution of novel biological features [16].

Standard phylostratigraphic classification based on protein similarity alone has several challenges. A much debated limitation is the difficulty of distinguishing orphan homologs of small, rapidly evolving genes from orphans of de novo origin [35, 36]. Another limitation is that phylostratigraphy infers gene ages based on negative evidence: the absence of a detectable, annotated, protein-coding homolog outside a clade. Thus, standard phylostratigraphy does not distinguish genes that are true orphans from those that are missing in related species due to bad genome assemblies or incorrect gene models.

An alternative approach to establish the de novo origin of a gene is to search for positive evidence of non-coding sequence in close relatives of the focal species. While in principle, this could be accomplished by simply searching the nucleotide sequence of the focal gene against whole genomes of related species, the large size of a genome and the often low-complexity of the novel gene, make false positives likely. A more powerful technique is to leverage syntenic data to identify the regions in the target genome where a homolog to each focal gene is expected to reside [7, 37]. By searching just this small region, the confidence that a similar sequence represents an ortholog is improved.

Syntenic analysis has provided a powerful approach to distinguish young genes with a de novo origin from genes encoding proteins which are unrecognizable in closely related species because they have undergone rapid evolutionary change [7, 37]. However, the use of synteny has been mostly limited to specialized, study-specific analyses [37] or to cases where tools are available for curated selections of genomes, such as the UCSC genome browser [25, 38]. Until now, no general genome-wide solution has been available for synteny-informed phylostratigraphy analysis.

Here, we present *fagin*, a new R package that generalizes, refines, and automates synteny-based phylostratigraphy. *Fagin* facilitates comparative analysis of genes across evolutionary clades, augmenting standard phylostratigraphy with a detailed, synteny-based analysis. Whereas standard phylostratigraphy searches the proteomes of related species for similarities to focal genes, *fagin* first finds syntenic genomic intervals and then searches within these intervals for any trace of similarity. It searches the (in silico translated) amino acid sequence of all unannotated ORFs as well as all known CDS within the syntenic search space of the target genomes. If no amino acid similarity is found within the syntenic search space, *fagin* will search for nucleotide similarity. Finding nucleotide sequence similarity, but not amino acid similarity, is consistent with a de novo origin of the focal gene. If no similarity of any sort is found, *fagin* will use the syntenic data to infer a possible reason. For example, *fagin* can detect indels, scrambled synteny, assembly issues, and regions of uncertain synteny.

*fagin* makes three major contributions to the phylostratigraphy field. 1) *Automation*. *fagin* offers the first automated package for synteny-based phylostratigraphy. 2) *Fine-tuned classification of query gene homologies*. By dividing homology inferences into three general classes (amino acid, nucleotide, and unknown), each with a set of subclasses, rather than using the typical binary classification (amino acid or nucleotide) for syntenic analysis, *fagin* provides a basis for assessing confidence in phylostratigraphy classifications and de novo designations. This makes *fagin* robust against bad data: genes in regions that are poorly assembled will fall into one of the Unknown-technical classifications. Also, if gene annotations are missing, matches against ORFs in the syntenic regions of the target genome will still be found (some of these matches may represent genes that are unannotated in the target genome; others may represent very rapidly-changing genes). 3) *Flexibility in (micro) synteny maps*. Whereas prior syntenic studies have been limited to synteny maps based on orthologous genes [37, 39], *fagin* can handle any synteny map, and is indeed particularly suited to micro-synteny maps produced by whole genome alignments. These fine-grained maps allow higher resolution through smaller inferred search intervals. They are also the basis for the inferred subclassifications.

As proof-of-concept, we explore the use of *fagin* in two cases studies centered on the focal species *Saccharomyces cerevisiae* and *Arabidopsis thaliana*. We systematically identify genes that that have arisen de novo from non-coding precursors and rapidly evolving genes that may have been missed by more traditional methods of gene annotation.

## Methods

**Fig. 1:**
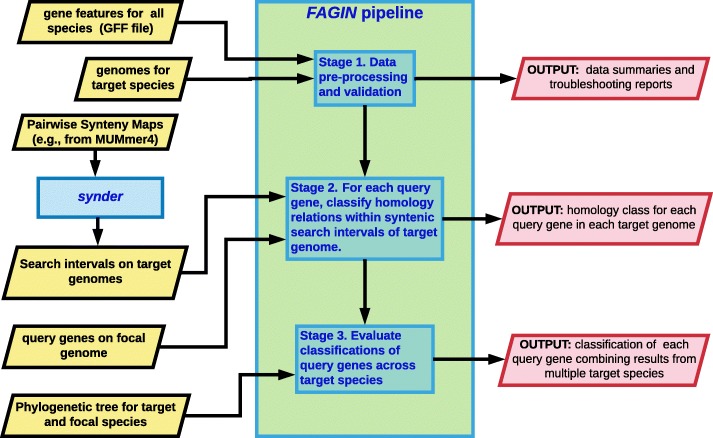
Overview of the *fagin* pipeline. Inputs (yellow rhombuses) are passed into *fagin*; the syntenic search intervals on the target genome corresponding to each query gene are delineated using synder [40]. The *fagin* pipeline consists of three stages. **Stage 1:** all input is validated, summarized, and secondary data (protein sequences, transcripts, ORFs) are extracted from genomes. **Stage 2:** The search intervals in the genomes of the target species that correspond to each query genes are searched to determine whether there is homology to the amino acid sequence (*AAic*) or nucleotide sequence (*NTic*) of that query gene, and if so where the homology occurs. Alternately, no homology might be detected (*unknown*). **Stage 3:** For each query gene, the homology classes are compared across the phylogenetic tree to infer that gene’s history. *Fagin* is customizable by the user. **Output** (red rhombuses) can include, e.g., summaries of the transformed input data, homology classes for each query gene against each target genome with statistical designations, and summaries of the homology results for each query gene across all genomes

The *fagin* pipeline can be sub-divided into three stages (Fig. 1): 1) pre-process input data; 2) search syntenic regions on target genomes for sequence similarity to query genes; 3) infer gene origins by comparing across genomes of related species. The entire pipeline is built using the rmonad pipeline package (available on CRAN). rmonad is designed to simplify the documentation, organization, benchmarking, and debugging of complex data analysis pipelines. Fagin uses parts of the external software [41–47] (See Additional file 1).

### Input data

The inputs required for *fagin* are: 1) a phylogenetic tree relating the focal species to one or more target species; 2) a genome sequence for the focal species and each target species; 3) Genome Feature Format (GFF) files that describes all gene models (or other features of interest) for each species; 4) the genes (or other features) to be queried from the focal species; and 5) pairwise synteny maps between the focal species genome and the genomes of each target species. These synteny maps are constructed from the genome pairs using an external software. For our case studies we used MUMmer4 [48] (for *S. cerevisiae* against other species in the genus) and Satsuma [49] (for *A. thaliana* against Brassicaceae relatives).

### Stage 1: pre-process input data and infer syntenic search intervals

In *Stage 1*, *fagin* cleans, validates, and summarizes all of the input data. The format of all input files are checked by *fagin*. Then, from the GFF files and genome sequences, *fagin* derives the protein sequences, transcript sequences, coding sequences (CDS), and the open reading frames (ORFs) on transcripts and whole genomes (see Additional file 1). The most difficult data pre-processing step is extracting gene models from the GFF files (see Additional file 1 for details). *fagin* also checks for signs of invalid input, such as stop codons appearing in the derived protein sequences. Then, *fagin* summarizes the assemblies and annotations of all genomes, the derived protein sequences, and the synteny maps.

*fagin* infers syntenic search intervals for each focal gene on each target genome, using input from the *synder* package [40]. *synder* traces each query gene on the focal species to a *search space* on the target genome, which is a set of one or more genomic intervals that are inferred to be orthologous. The purpose of delineating search intervals is to winnow false positives and increase sensitivity by limiting the search to orthologous regions of the target genome. In Stage 2, these syntenic search intervals are analyzed to find traces of homology to the CDS of the query gene.

### Stage 2: determine homology classes of each query gene in the search interval of each target genome

**Fig. 2:**
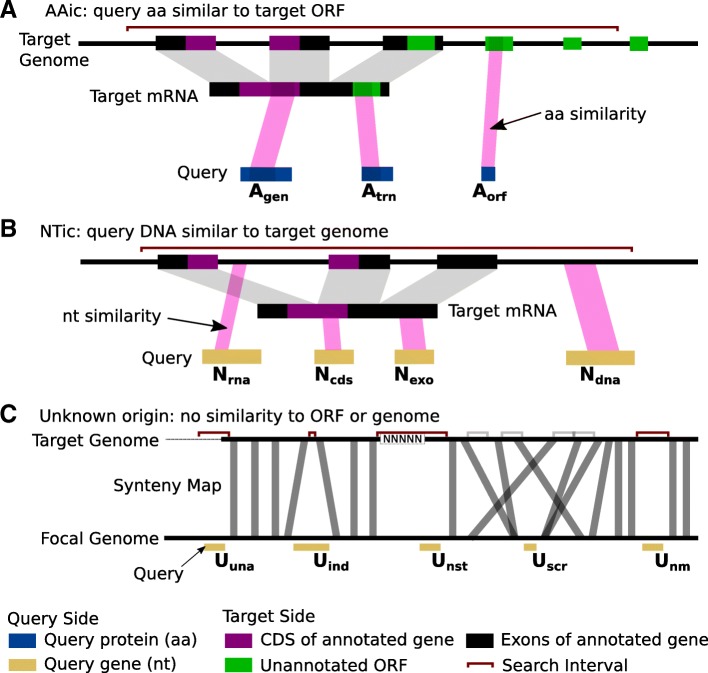
*fagin* classifies the genomic context. F*agin* infers genomic context of query genes or other genomic features on the focal genome by searching for homologous sequence within syntenic search intervals on the target genome. For protein-coding query genes, *fagin* searches for homology to the protein (aa)(**a**) or entire sequence (nt) (**b**) of the query gene. It also categorizes the unknown (**c**). Grey bars in **C**, syntenic links. The *fagin* classification is indicated below each query, in bold black font. Rooting the homology searches to the syntenic regions narrows the search space, thereby increasing the sensitivity

**Fig. 3:**
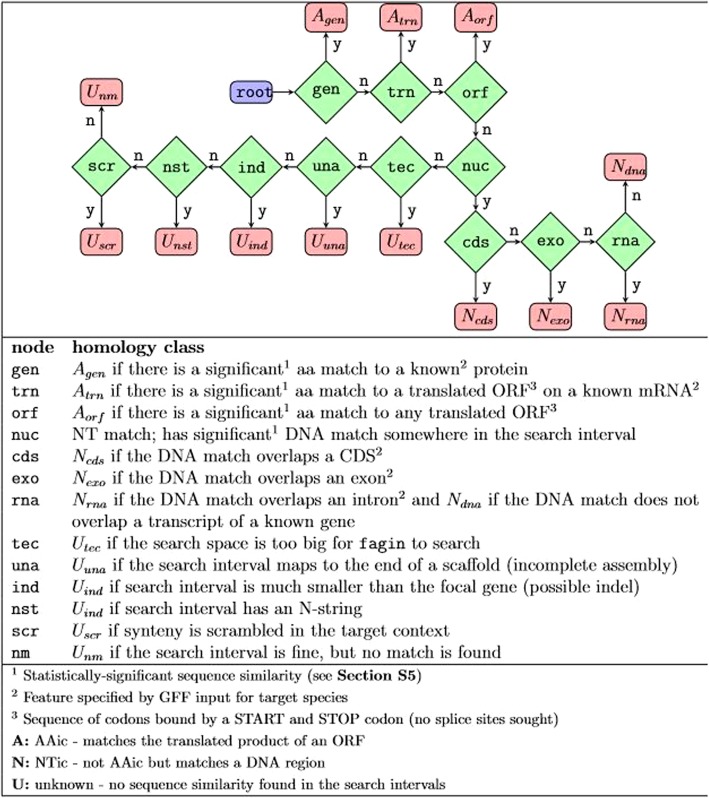
The default *fagin* decision tree for determining homology classes. The process first asks whether the focal gene has a significant aa match to an annotated protein in the synder-derived search interval of the target genome (green diamond node, gen). If yes, the gene is classified as *A*_*gen*_, otherwise, the next question is asked. This process continues along the decision tree until a homology class (red, rectangular leaf node) is assigned. y, yes; n, no. The tree can be modified or replaced by the user. For example, nodes with other evidence (e.g. proteomic, transcriptomic) or analysis can be added, with associated homology

In *Stage 2*, each query gene is assigned, relative to its inferred search intervals, to a homology class (Fig. 2). By default, *fagin* considers three general cases (Fig. 3): **AAic** if there is aa similarity between the protein encoded by the query gene and the translation product of a known CDS or any other ORF within the search interval; **NTic**, if there is nucleotide similarity of the query gene to any nucleotide sequence (transcript or genomic) within the search intervals; and **Unknown** if no similarity can be found, in which case *fagin* will attempt to determine the biological or technical reason why no similarity was found (Fig. 2). The assignments are made by following a binary decision tree (Fig. 3). This tree may be customized. Here, we focus on the default tree of *fagin*. The default ORF cutoff length is 30 codons and the default *p*-value threshold for matches is 0.05, after statistical adjustments.

#### AAic class

The amino acid sequence encoded by each query gene is searched against the translated CDSs and ORFs of the syntenic search intervals in each target species to infer the presence or absence of a potential ortholog (Fig. 2). Following the decision tree, *fagin* divides the AAic class into three groups. A query gene is classified into the first affirmative case on the decision tree (see Fig. 3).

The query gene is *A*_*gen*_ if the encoded protein of a query gene has amino acid similarity to an annotated protein of the target species that overlaps a syntenic search interval. This class is strong evidence that a query gene has an ortholog in the target. The next two classes, *A*_*trn*_ and *A*_*gen*_ are amino acid matches to *potential* coding sequences. *A*_*trn*_ indicates similarity to an ORF (other than the CDS) on an annotated mRNA, for example, a short ORF in the 3′ UTR. *A*_*orf*_ indicates similarity to a translated ORF that does not overlap an annotated mRNA. *A*_*orf*_ is an expected class for unannotated orthologs, rapidly-changing genes, and also potential de novo orphans (A researcher could test among these possibilities by comparison of similarity distribution, analyses of within species genomes, further RNA-Seq data, proteomic data, and experimentation).

#### NTic class

If a query gene has no amino acid similarity to any CDS or ORF overlapping its target-side search interval, then evidence for nucleotide matches is sought. A focal gene is classified as *N*_*cds*_ if it contains a DNA match to a CDS that overlaps the target-side search interval (Fig. 2, Fig. 3). A query gene is classified as *N*_*exo*_ if it contains a DNA match to an exon that overlaps the target-side search interval. A query gene is classified as *Nrna* if it contains a DNA match to an intron of any target gene that overlaps the search interval. Finally, a query gene is classified as *N*_*dna*_ if it contains a DNA match anywhere within its search interval that does *not* overlap any known gene; *N*_*dna*_ is an intergenic match.

Since NTic query genes have no amino acid similarity to any ORF in the search interval (a similarity would have led to an AAic classification), then the ortholog of the NTic focal gene is likely non-genic. NTic classifications are thus consistent with a de novo origin.

#### Unknown class

If the query gene has no significant amino acid or nucleotide similarity within its target-genome search interval (i.e., the query gene has an Unknown origin), then *fagin* will search for the most likely reason why no similarity was found. As with AAic and NTic classes, a query gene is classified into the first affirmative case on the decision tree (Fig. 3).

Several cases are biologically interesting (Fig. 2c, even more so in comparison to the analogous results for conserved genes (see Results). The query gene is *U*_*ind*_ if its search interval on the target genome is much smaller than the query gene. This implies the ortholog may have been either deleted in the target genome or inserted in the focal genome (i.e., an indel). The query gene is *U*_*scr*_ if the order of elements in the chromosome near the focal gene is highly scrambled relative to the target genome. If the species provided to *fagin* are too distant for synteny to be conserved, then most genes will fall into this category; however, in near relatives with generally conserved synteny, this might indicate a region of high chromosomal instability. The query gene is *U*_*nm*_ if it is in a syntenic region that is large enough to accommodate it, but no match is found. This could be due to a rapid mutational evolution such that the gene that can no longer be detected even with the reduced search space and high resolution of *fagin*, or due to the gene having been translocated out of the region, perhaps with a transposon.

Several of the U classifications are due to technical aspects associated with the genome annotations or assemblies, or rarely to the current inability of *fagin* to search very long search spaces. The query gene is *U*_*una*_ if it is inferred by synder to be in a search interval that is flush against an end of the scaffold of the target genome. This implies that the ortholog in the target genome may be missing from the target genome assembly. The query gene is *U*_*nst*_ if the search interval in the target genome contains a string of unknown bases (N characters). This is also a sign of an incomplete assembly. The query gene is *U*_*tec*_ if any search interval was skipped because it is too long for a *fagin* search. The current release of *fagin* relies on a Smith-Waterman alignment to determine similarity scores. The runtime of this algorithm increases with the product of the focal and target lengths. To avoid extremely long runtimes, *fagin* has a cutoff for the largest space it will search. If many genes are classified into this category, then the user should increase the maximum search space threshold or modify *fagin* to use a faster algorithm. Membership in the *U*_*tec*_ category was almost non-existent for our two case studies.

### Stage 3: determine origins

**Fig. 4:**
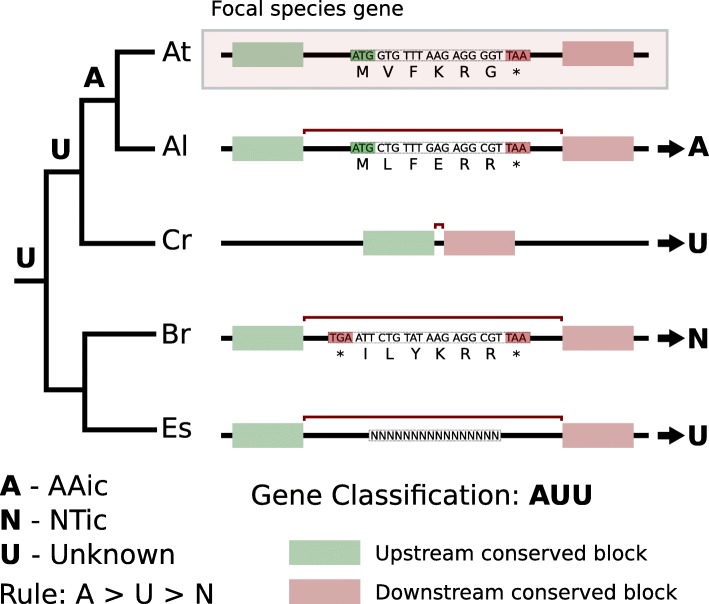
Synthesizing syntenic analysis information across target genomes: UNA classes. On the **left** is the Brassicaceae family tree. On the **right**, is the syntenic context for an imaginary query gene versus each target species. The query gene matches a gene in the most closely related target species, so is classified as AAic (A). In Cr, there is evidence of an indel, but no positive evidence for presence/absence of the gene in the species, so it is labeled as Unknown (U). The most distant branch from the focal species contains two species, one of which contains a positive NTic match and for other data is possibly missing. Since it is possible that an AAic match exists in Es, the branch is classified as Unknown overall. These three labels (A, N, and U) are a qualitative indication of the support for a gene being present along each cousin branch. The query gene is certainly not an orphan gene, but more precise statements are not justifiable. Including more target species could support a stronger inference for the query gene origin. This figure illustrates comparison at the species level. As more sub-species genomes are sequenced, we recommend inclusion of lineages within the focal species for a more powerful analysis, and for improved ability to distinguish rapidly evolving orphans from de novo orphans

In *Stage 3*, the assignments of each query gene from *Stage 2* are used to assign a phylostratum for each gene and to evaluate the level of support for the assignments. In the default settings of *fagin*, a potential biological origin for each query feature is inferred by a “UNA” classification, based on the assignment of the query feature to Unknown, NTic, and/or AAic classes across lineages (Fig. 4). The UNA classes collate information from across the tree into a single vector of labels representing level of support for the existence of a genic or non-genic homolog in each outgroup (i.e., one label for each node from focal species down to the root of the tree).

The query genes are assigned to UNA classes as follows. Let internal nodes from the focal species to the root of the species tree be numbered *p*_0_ to *p*_*K*_, where *p*_0_ is the parent of the focal species, *p*_1_ is the grandparent, and so on down the trunk of the tree to the root, *p*_*K*_. Borrowing genealogical terminology, a set of “cousin” species can be defined for each ancestor (*p*_*i*_) of the focal species. The 0th cousins, *t*_0_, (i.e., siblings) are the species descending directly from *p*_0_. The 1st cousins, *t*_1_, are the species descending from *p*_1_, and so on to the *K*th cousins. The goal is to determine which ancestor, *p*_*i*_, first possessed the gene in a coding state; that is, find *i* where the ancestral species *p*_*i*_ has a protein-coding homolog of the query gene but where *p*_*j*_ does not for all *i < j* ≤ *K*.

For each ancestor, *fagin* infers whether an orthologous coding gene could have existed. To this end, we collapse the homology class of each species tree, from *t*_0_ to *t*_*K*_, to a single homology class. If we assume that the event leading to the origin of the ancestor of the focal species gene occurred only once (i.e., a single-birth model), then AAic classes should appear only in cousins descending from the ancestor that had the original gene. Under this assumption, if *any* leaf in the *t*_*i*_ tree is classified as AAic, then the entire subtree is classified as AAic. If *all* leaves in the subtree are NTic, then the subtree is classified as NTic. In cases in which the leaves include at least one unknown and zero or more NTic, the entire tree is classified as unknown, since the unknown gene *could* be AAic. This is a stringent rule that is biased to a high estimation of uncertainty.

In summary, the subtree classification rule is

1 $$ {S}_{ij}=\left\{\begin{array}{l}A\kern1em \mathrm{if}\kern0.5em \mathrm{any}\kern0.5em \mathrm{leaf}\kern0.5em \mathrm{in}\kern0.5em {t}_i\kern0.5em \mathrm{is}\kern0.5em \mathrm{AAic}\kern0.5em \mathrm{relative}\kern0.5em \mathrm{to}\kern0.5em \mathrm{the}\kern0.5em j\mathrm{th}\kern0.5em \mathrm{focal}\kern0.5em \mathrm{feature}\\ {}N\kern1em \mathrm{if}\kern0.5em \mathrm{all}\kern0.5em \mathrm{leaf}\mathrm{s}\kern0.5em \mathrm{in}\kern0.5em {t}_i\kern0.5em \mathrm{are}\kern0.5em \mathrm{NTic}\kern0.5em \mathrm{relative}\kern0.5em \mathrm{to}\kern0.5em \mathrm{the}\kern0.5em j\mathrm{th}\kern0.5em \mathrm{focal}\kern0.5em \mathrm{feature}\\ {}U\kern1em \mathrm{otherwise}\end{array}\right. $$

Where *s*_*ij*_ is the label assigned to the *i*th cousin subtree (or the *i*th position in the UNA vector).

Following this pattern, a UNA classification, a vector of length *K* + 1, can be inferred for each subtree (see Fig. 4). The gene can be classified into a synteny-based phylostratum for gene *i* by finding the maximum *i* such that s_ij_ = A and s_zj_ ≠ A ∀*i* < *z* ≤ K. For example, if there is support across all nodes for the AAic class, from siblings to most distant cousins, we can infer that the earliest common ancestor was genic.

Alternate ways to infer the origin of gene features based on multiple target genomes are possible, and can be customized in *fagin*. For example, the classification could take into account the length of the matches to the target genome ORF, or it could incorporate the sub-classifications of AAic, NTic and Unknown.

This approach to inferring gene origin can be considered a significant modification of standard phylostratigraphy. In standard phylostratigraphy, the proteomes of related species are searched for similarity to a focal gene. If a significant hit is found, the species is classified as having a homolog. This classification is similar to the *fagin* AAic classification, *except* that in *fagin*: 1) the search is restricted to *syntenically* matching regions; and; 2) the amino acid hits may correspond to annotated CDS, unannotated ORFs on known mRNAs, or unnannotated mono-exonic ORFs anywhere in the search interval; and 3) a distinction is made between classifications based on positive evidence (i.e., A or N) and those based on negative evidence (U).

Thus, whereas standard phylostratigraphy is based on a binary decision about the presence or absence of a homolog [32], and synteny-based de novo gene pipelines classify the *matches* in the syntenic search interval (e.g., [37, 39]), *fagin* is based on a three-way decision, followed by subclassifications: 1) a possible protein-coding match; 2) positive evidence that there is *no* protein-coding match; and 3) no answer can be found. Essentially, standard approaches merges the *fagin* categories N and U, and thus does not distinguish between matches that are missed due to bad data and matches that are missed due to absence of the gene.

## Results

We demonstrate use of the *fagin* pipeline on two focal species: *S. cerevisiae* and *A. thaliana*. These species have been analyzed using standard phylostratigraphy in earlier papers identifying 423 Saccharomyces-specific genes [50] and 2425 Brassicaceae-specific genes [51]. Building off these prior studies, clade-specific genes were fed into the *fagin* pipeline for deeper analysis. Both focal species have good genome assemblies, but the target species in each study were of variable quality (see Table 1). We built pairwise synteny maps between the focal genomes and each target using MUMmer4 (for *Saccharomyces*) and Satsuma [49] (for *Brassicaceae*). The synteny maps are fairly dense, with several hundred blocks per megabase and block length medians ranging from 102 to 389 (see Additional file 1).

**Table 1:** Genomic statistics for species in the Brassicaceae (top) and Saccharomyces (bottom)

species	nseq	n50 (nt)	size (nt)	prots	Ns
*A. thaliana*	7	23,459,830	119,667,750	35,386	185,738
*A. lyrata*	695	24,464,547	206,667,935	32,550	22,960,134
*C. rubella*	773	15,040,190	133,063,876	28,713	3,314,705
*E. salsugineum*	638	13,441,892	243,110,105	29,485	4,665,582
*B. rapa*	40,249	26,286,742	284,129,391	51,005	10,904,295
*S. cerevisiae*	17	924,431	12.2 M	6008	0
*S. paradoxus*	832	49,124	11.9 M	5933	0
*S. mikatae*	1648	20,026	11.5 M	6086	0
*S. kudriavzevii*	2054	11,253	11.2 M	6529	2127
*S. arboricola*	35	879,294	11.6 M	3659	224,325
*S. eubayanus*	24	896,107	11.7 M	5379	121,986
*S. uvarum*	1098	25,082	11.5 M	5721	0

*fagin* first infers homology classes between two species. From the homology classes, we infer the phylostrata for each focal gene and compare them to those inferred through standard methods. Finally, we break the phylostrata into finer classes based on UNA vectors.

### Homology classes

**Fig. 5:**
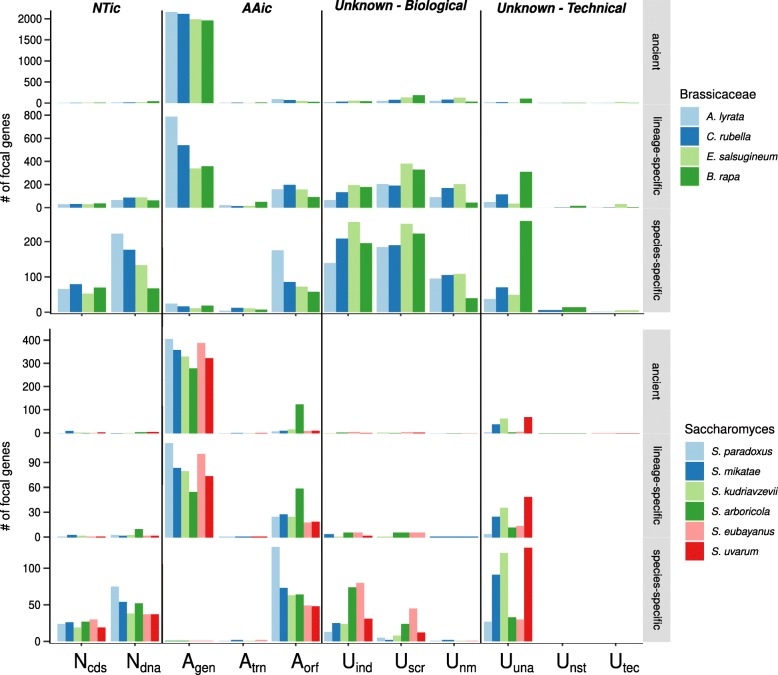
*fagin*-inferred homology classes for the Brassicaceae (above) and Saccharomyces (below) case studies. In each study, the top row of plots, labeled ancient, represents a random sample of genes from ancient strata (outside Brassicaceae or Saccharomyces). The **lineage-specific** row includes genes that are unique to the clade, but that are not unique to the focal species. The **species-specific** row includes only the orphan genes. The original inferences of orphan, lineage-specific, and ancient genes were made by standard phylostratography [50]. Each group of bars represents the number of query genes that fall into a given homology class in relation to each target species

The homology classes for the Saccharomyces and Brassicaceae studies are summarized in Fig. 5. Summaries of the search interval lengths and inferences about syntenic ambiguity or genome assembly issues is available in Additional file 1. In each study, all orphan genes, all lineage-specific genes (unique to genus for Saccharomyces, unique to family for Brassicaceae), and a random sample of ancient genes, as inferred by standard phylostratigraphy [50], were passed through the *fagin* pipeline. As expected, the majority of the ancient genes fall into the AAic class (see the **ancient** rows of bar plots in Fig. 5). However, about 20% of ancient *S. cerevisiae* query genes are classified as *A*_*orf*_ relative to *S. arboricola*. This implies a strong disagreement between the gene annotations in the focal species, *S. cerevisiae*, and the *S. arboricola* target species; indeed, only 3659 genes are annotated in *S. arboricola* (Table 1).

**nseq**, number of scaffolds in the assembly; **n50**, number of bases in the scaffold that contains the genomic midpoint in a list of scaffolds sorted by length; **size**, size of the genome; **prots**, number of gene models in the genome; **Ns**, number of unknown bases (N) in the genome assembly.

In both case studies, a high proportion of the orphan genes are classified into the “Unknown-Technical” category, predominantly *U*_*ind*_ (indels), *U*_*scr*_ (syntenically scrambled), *U*_*una*_ (bad assembly), and *U*_*nm*_ (no match found). These subclassifications can be informative. For example, the analysis provides an alternative approach to assess poor genome quality. For example, about a third of the *A. thaliana* genes classified as orphans have no syntenic region in *B. rapa*, and are inferred as “missing due to bad assembly”. This finding reflects that the *B. rapa* genome assembly we used for this species is very incomplete (see Table 1).

Quite interesting are the many genes from *A. thaliana* (and to a lesser extent, *S. cerevisiae*) inferred by phylostratigraphy to be “orphans”, which upon syntenic analysis by *fagin* fall into an “Unknown-Biological” category: (*U*_*ind*_(indels), *U*_*scr*_ (syntenically scrambled), or *U*_*nm*_ (no match found). This contrasts with the over 90% of ancient genes that have syntenic counter-parts in all target species. These vastly different assignments of the orphan versus ancient genes suggest the intriguing possibility that orphans are less likely to be associated with a syntenic region because they arise in regions of genomic perturbation.

### Synteny-based phylostratigraphy

**Fig. 6:**
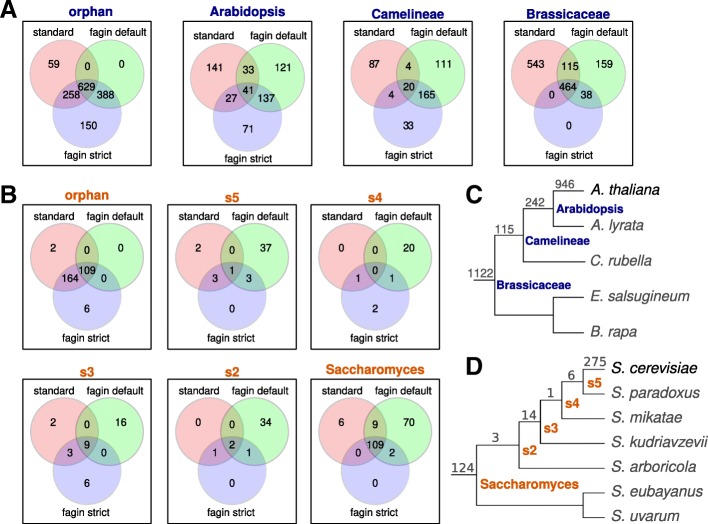
Comparison of assignments in gene classifications by three methods. The *Brassicaceae* study (**a**) represents overlaps in gene classifications across four phylostrata of *Brassicaceae*. The Saccharomyces study (**b**) represents overlaps in gene classifications across six phylostrata, from the *S. cerevisiae*-specific **orphan** phylostrata, through the genes unique to each of the **s5** to **s2** internal clades, to the genes conserved across the *Saccharomyces* genus. The three methods of comparison are 1) **standard** which represents standard phylostratigraphy; 2) **fagin default** which is the default *fagin* behaviour of identifying phylostrata based on presence/absence of any AAic inferred ortholog; and 3) **fagin strict** which identifies phylostrata based on presence/absence of amino acid matches only to annotated target genes (similar to standard phylostratigraphy). All methods use the set of protein coding genes that were inferred through standard phylostratigraphy to be limited to the Brassicaceae or Saccharomyces clades. **c** and **d** are the species trees representing the target genomes used for Brassicaceae [52] and Saccharomyces [53], respectively. The numbers indicate the number of genes in each clade according to standard phylostratigraphy (from phylostratr for Saccharomyces [50]; [51] for Brassicaceae). Nodes on the Saccharomyces tree, orange text, are labeled as s5-s2 because there are no taxonomic names for these within-genus clades

We compare standard phylostratigraphy results to two *fagin*-based approaches (Fig. 6). The first *fagin*-based approach, *fagin*-default, infers homologs in target species based off all three AAic classes. The second *fagin*-based approach, *fagin*-strict, infers homologs based only on matches of the query amino acid sequence to known protein-coding genes in the target species (the *A*_*gen*_ class); this roughly emulates standard phylostratigraphy but is limited to syntenic genes.

In the Saccharomyces study (Fig. 6b), the *fagin*-strict classifications agree closely with standard phylostratigraphy. However, *fagin*-default infers older origins for 164 genes that were classified as orphans by standard and *fagin*-strict. The majority of these genes were inferred as being older by *fagin*-default due to an amino acid match of the query gene to the amino acid predicted sequence of an unannotated non-genic ORF (*A*_*orf*_). There are two interesting interpretations. 1) An orthologous gene might be located in the syntenic region of the target species but it might not be annotated as a gene. In this case, the gene would not be an orphan, but rather would be older. Standard phylostratigraphy would not detect these homologs since only annotated genes are searched. 2) The match might be to the predicted amino acid sequence of an untranscribed and/or untranslated ORF. In this case, the query gene might be a very-rapidly-evolving orphan, i.e., an orphan that did not originate de novo during or post-speciation. It is difficult to detect genes that are rapidly changing, and the mechanisms for this change are also interesting [54]. Possible methods to gain insight into which target-side ORFs are real hits would be to compare the size of the ORF to the ORF of the focal gene, to assess evidence of transcription and translation, and to look for evidence of selection.

Among the *S. cerevisiae* orphan genes, one gene is unique to standard analysis and six are unique to *fagin*-strict. The gene uniquely designated by standard phylostratigraphy as an orphan is possibly a case where the reduced search space, and resulting higher statistical resolution, led to an inferred homology that could not be detected in the standard phylostratigraphy search against the full target proteome. The genes uniquely designated as orphans by *fagin*-strict could be genes that hopped out of context (e.g., transposed) and were thus absent from the syntenic search space. A search for transposon footprints might reveal if this was the case.

In the Brassicaceae study, the standard and *fagin*-strict methods gave very different results. Since the main difference between the two methods is that *fagin*-strict is limited to searching syntenic genes, the search intervals inferred from the synteny maps must be missing many of the true orthologs. *Fagin*-based phylostratigraphy treats genes that cannot be found as genes with no homologs. Many of the query genes have Unknown homology-class across all target species and are thus classified as orphans. Thus the genes of unknown original and the genes of confirmed recent origin are pooled. To resolve these groups, one could look deeper into the gene classifications *fagin* provides.

### Finer grain analysis of phylostrata with UNA classes

The homology classes contain much information that is lost when reducing down to just phylostratigraphy labels. We can gain more insight into the support for the phylostrata classes by looking at the *UNA* vectors (see Additional file 1). A summary of UNA classes for the Brassicaceae study is shown in Table 2. This table partitions all the genes in the focal species into the four phylostrata as well as a fifth class where there is no evidence for a syntenic homolog even in the closest relative.

**Table 2:** UNA labels for Brassicaceae ordered by phylostratum

Class	Brassicaceae-specific	non-Brassicaceae-specific	phylostratum
AA**A**	474	2033	
AN**A**	52	7	
UA**A**	26	18	
AU**A**	44	44	
NA**A**	18	4	Brassicaceae
NN**A**	34	0	
NU**A**	3	3	
UN**A**	8	0	
UU**A**	26	14	
A**A**U	194	111	
A**A**N	20	3	
N**A**N	7	0	Camilineae
N**A**U	27	3	
U**A**U	27	17	
**A**NN	38	0	
**A**NU **A**UN	47 5	6 0	Arabidopsis
**A**UU	256	58	
NNN	48	1	
NNU NUN	80 4	0 0	*A. thaliana*
NUU	162	4	
UNU	25	0	Unknown
UUU	800	99	

Among the Brassicaceae-specific genes as inferred by *fagin* (Table 2, *Brassicaceae*-specific), 474 are genes classified as AAA. These are genes with strong positive evidence of being present across the Brassicaceae clade. In contrast, the 34 NNA are possibly orphan genes, in which the deepest A is likely a false positive, such as a match to a non-genic ORF that is actually non-functional. The 26 UUA genes are of unclear phylostrata, with weak evidence for their Brassicaceae-spanning classification. Incorporating additional genomes into the analysis might help resolve these disparities.

The 48 NNN query genes are the most strongly supported de novo orphan genes. The 162 NUU, 80 NNU, and 4 NUN genes are also supported de novo orphans, for which analysis of more target genomes could provide more support. A particularly interesting class of genes are the lineage-specific genes of de novo origin with the labels ANN and AAN. These are genes with positive evidence of being de novo, having evolved from non-genic precursors and survived to spread across several species. These de novo genes could be studied to shed light on the dynamics and evolution of the functional evolution of de novo genes.

The **Brassicaceae-specific** column contains counts of query genes with each UNA label from among genes that is inferred by standard phylostratigraphy to be Brassicaceae-specific. The **non-Brassicaceae-specific** column contains counts of older genes that are used as a control. The **phylostratum** column contains the phylostratum as inferred by the deepest character in the UNA vector that is AAic (the **A** in bold).

The 800 UUU genes are genes with no positive evidence of being present in any form outside *A. thaliana*. Standard phylostratigraphy did not detect them in any species, and they have no syntenic homologs. All these are candidate orphans. F*agin* can offer hints about the origin of these UUU genes, from their synteny-based U sub-classes. A deeper look into the sub-classes, and further analysis of the search intervals, could give us a better understanding of the origin of each of these genes. Some may be missing for technical reasons (incomplete assemblies) while others may be missing for more interesting biological reasons (rapid syntenic rearrangements or transposition).

The UNA classes can of course be further broken down on a gene-by-gene basis into the homology classes. The actual alignments from all the homology searches is stored by *fagin*. All of this data can serve as a starting point for deeper analysis of the origins of specific genes (see Additional file 2 and Additional file 3).

## Discussion

A key difference between synteny-based phylostratigraphy and standard phylostratigraphy is the emphasis on positive evidence [37, 39]. The methods differ in two significant ways. First, the synteny-based approach is more sensitive, since it searches the small, synteny-based search space, instead of the entire proteome. This effectively leads to younger classifications. Second, by limiting the search to syntenic regions, it both avoids false positives and, when synteny is unclear, misses true positives ─ in either case, the synteny-based approach infers younger classes. However, analysis of gene age based on synteny has the limitation that it is restricted to only those cases when synteny is reasonably conserved. For example, synteny may be sufficiently conserved across the primate family, but probably not across Animalia. In this sense, syntenic analysis uncovers only recent evolutionary events. Similarly, a target genome may have undergone multiple rearrangements, and for some regions of that genome no syntenic region might be identifiable. Thus, each method has different strengths and weaknesses. We propose that it is important to use both methods ─ *genome-wide phylostratigraphic analysis in combination with more in-depth syntenic analysis* ─ to gain the best understanding of the evolutionary trajectory of each gene.

Those genes designated by standard phylostratigraphy as “orphans” but that have no observed synteny to a region in a sister genome are interpreted differently by different researchers. In some studies, they are relegated explicity or implicitly, to the large group of “genes of unknown origin”. Other studies classify all genes with no detectable homology in related species as orphans (i.e., studies in which the classification “orphan” depends solely on absence of significant similarity to an annotated protein). Still other studies have categorized the genes with no syntenic matches as not being de novo orphan genes (e.g., [38]). In actuality, *if a gene cannot be traced, the origin is unclear*. With *fagin*, although no origin is assigned to genes without syntenic matches in a target species, these genes are further classified as non-syntenic for “biological reasons” (such as deletion events or evolution beyond recognition) or non-syntenic for “technical reasons” (such as missing sequence or poor assembly). Thus, *fagin* subcategories resolve genes with no syntenic match into specific inferred phenomena, such as deletion/insertion events, missing sequence or poor assembly.

Synteny provides an important tool identify genes of de novo origin [7, 25, 37, 38]. *fagin* makes the method automatic, general, and reproducible. Further, it extends the technique, by offering a deeper analysis of the source and magnitude of the classification error. *Fagin* can be applied to annotate orphans of de novo origin in new genome sequencing projects, to identify promising orphan gene candidates for further experimental research, and to directly study the dynamics of de novo gene evolution. Likewise, it can provide candidates for proteins that are targets of ultra-rapid evolution-beyond-recognition. For example, those genes that are classified by standard phylostratigraphy as orphans, but reveal an amino acid match to the CDS of a known gene in the more sensitive search to a syntenic interval of a target genome, are candidates for being ultra-rapidly changing genes. Likewise, inclusion in-species lineage could help to provide positive evidence identifying rapidly-changing orphan genes.

*fagin* differs from current syntenic approaches in four main ways. First, it enables a user to seamlessly go from data input to final results and summaries. Second, the *fagin* pipeline is flexible and easily modified. A user can compare various methods for classification of the same genome data sets, or evaluate classifications based on different determinations of the syntenic search space used for each query gene. A user also can choose to extract intermediate data from any step in the pipeline. Third, *fagin* classifies every gene by probing the syntenic space of each query gene and explicitly distinguishing among query genes that have an amino acid match, those that have only a nucleotide match, and those that have no match, i.e., are of unknown origin. These classes are then sub-categorized, inferring extensive information about each gene’s origin. Finally, *fagin* uses multiple target genomes, thus providing additional evidence to support query gene classifications. These features help to highlight the ambiguity of assignments, and the challenges of working with complex biology and incomplete data.

*fagin* can also be used to study overprinting, the phenomenon in which a single gene encodes more than one protein or one reading frame gives way to another over evolutionary time. Overprinting is a common scenario in viruses, in which many such overprinted proteins are orphans [55, 56]. Though less studied, overprinting also occurs in Eukaryotes [57] and may be involved in de novo gene origin [6]. The signature of an overprinted gene in *fagin* would be a query gene that does not match any annotated (target-side) syntenic coding gene but that does match a transcribed ORF that overlaps a known gene (i.e., *N*_*cds*_ class).

*fagin*’s consideration of multiple genomes facilitates comparisons of orthologs across evolutionary time. Specifically, *fagin* will allow for the systematic identification and study of lineage-specific genes of de novo origin that are conserved across a subclade, but are shown by syntenic analysis to be derived from non-genic sequence outside that subclade. Subclades can encompass within-species populations. Since these lineage-specific de novo genes have homologs, they can be studied in their evolutionary context. Analysis of the sequence of these de novo genes in related lineages will shed light on how they evolved. Do they specialize their expression patterns and functions in different lineages? How does their disappearance/deletion rate compare to that of older genes? Do they become longer and more complex over time? Do their codons become more optimized? How do the properties of these genes change is relation to those of rapidly evolving genes of more ancient origin? By automating the complex process of syntenic phylostratigraphy, *fagin* will allow such studies to be done on a large scale. This would be a four-step process: 1) collect data for all members of each focal and target genome in a clade; 2) construct pairwise synteny maps between focal and target genomes; 3) run a standard phylostratigraphy study (this may be automated with phylostratr [50]); and 4) run the lineage-specific genes through *fagin*.

*fagin*’s generalizable structure greatly simplifies additions and extensions. In particular, the flexible decision tree is foundational to *fagin*. The decision tree for determining homology classes can be altered by adding additional nodes that contain different data-types or rules. The structure of this tree is central to simplifying writing extensions and making changes. *Fagin* could be merged with phylostratr to integrate synteny-based phylostratigraphy for shallow clades with standard phylostatigraphy for deeper clades. The tree could be adjusted to follow the analysis pipeline suggested in [39]. Transcriptomics data indicating which ORFs are transcribed in the focal and target genomes, could be added to *fagin*, as could evidence of translation, such as ribosome footprinting or proteomic mass spectroscopy dictions of unannotated, spliced, transcripts. Adding new nodes to the decision tree would also add new classes of orthologs in the target genome, with richer information and support. For example, adding a new node for transcriptomic data and one for proteomic data would provide two new AAic classes of orthologs: one for unannotated ORFs with experimental evidence of transcription, and one for unannotated ORFs with proteomic support.

## Conclusion

The *fagin* R framework-based software extends flexible, modular phylostratigraphy with an automated, customizable, and detailed contextual analysis. As such, it supplies a synteny-based pipeline to explore gene evolution, augmenting standard phylostratigraphy by determining the genomic context of each query gene in a focal species, as compared to homologous sequence in target species. We anticipate that *fagin* will serve as a general framework for phylostratigraphy and orthology inference, providing a consistent and reproducible way to compare mechanisms of evolutionary change across genomes. Since *fagin* relies on synteny, it will become increasingly useful as the number and quality of genome sequences rises.

## Additional files

Additional file 1: Supplementary Material. (PDF 154 kb)

Additional file 2: Supplementary files saccharomyces. (XLSX 618 kb)

Additional file 3: Supplementary files brassicaceae. (XLSX 2867 kb)

## Data Availability

The code for running the case study is available at https://github.com/arendsee/fagin-case-studies. All data needed for the yeast case study (https://datahub.io/arendsee /fagin-yeast) and the Brassicaceae synteny maps (https://datahub.io/arendsee/brassicaceae-synmaps) are available on DataHub. The yeast genomes, tree, and annotations were retrieved from the Saccharomyces Genome Database [58]; Arabidopsis annotations are from TAIR (https://www.arabidopsis.org/). Summaries of the case study results are given in the Additional file 2 and Additional file 3 (both of which are automatically generated in the scripts provided in the case study code).
