# Supplementary material for: fagin: synteny-based phylostratigraphy and finer classification of young genes
Source: BMC Bioinformatics. 2019 Aug 27;20:440. doi: 10.1186/s12859-019-3023-y (PMC6712868; doi:10.1186/s12859-019-3023-y)
Supplement: Supplementary file 1 — Supplementary Material. (PDF 154 kb) [file 12859_2019_3023_MOESM1_ESM.pdf]

Supplementary Material

S1 Synteny map construction and summaries

Table S3: Numeric summary of the lengths of the syntenic blocks (in nucleotides) in the synteny maps between the focal species (*A. thaliana* and *S. cerevisiae*) and each of the listed target species for the Brassicaceae (**top**) and Saccharomyces (**bottom**) case studies. **N**, total number of blocks in the synteny map.

	Synteny Blocks (nt)					
Species	min	q25	median	q75	max	N
A. lyrata	25	33	163	365	9280	229562
C. rubella	25	39	197	399	6264	151688
L. rapa	25	150	214	366	10260	195085
E. salsugineum	25	146	222	400	8248	131874
S. paradoxus	63	177	389	852.00	13716	12232
S. mikatae	63	80	275	552.25	6836	6688
S. kudriavzevii	65	75	257	489.00	4870	5189
S. arboricola	65	71	159	417.00	4103	4534
S. eubayanus	65	70	102	375.00	4751	3914
S. uvarum	65	70	104	376.00	6836	3833

S2 `synder` results and summary

Table S4: Numeric summary of the lengths of the search intervals inferred by `synder` [38] between the focal species (*A. thaliana* and *S. cerevisiae*) and each of the listed target species for the Brassicaceae (top) and Saccharomyces (bottom) case studies. Column 2-7 refer to the minimum, 25th quantile, median, 75th quantile, and maximum of the block lengths. The final column, **N**, is the total number of blocks in the synteny map.

	Search Intervals (nt)					
Species	min	q25	median	q75	max	N
A. lyrata	1	807	1654	2949	2909847	72713
C. rubella	2	1040	2004	3440	216346	64133
S. rapa	1	826	1737	4057	298748	148594
E. salsugineum	2	1150	2299	4617	900973	66102
S. paradoxus	1	670.0	1215	2211	26047	13219
S. mikatae	1	556.5	1714	3858	31027	16039
S. kudriavzevii	1	1006.0	2332	4919	26409	21460
S. arboricola	1	1956.0	4643	9723	51035	19660
S. eubayanus	3	2480.0	5967	12521	61711	23995
S. uvarum	1	1327.5	3419	7746	45931	26095

Table S5: Summary of `synder` [38] flags for the Brassicaceae (**top**) and Saccharomyces (**bottom**) case studies. **Between** means that the query gene was between but did not overlap any synteny block. **Lo**, **Hi**, **Both**, and **None** relate to whether the edges of the search interval are in a syntenically unambiguous region. A gene is counted as **Lo** if any search interval lower bound is unambiguous, **Hi** is any upper bound is unambiguous, **Both** if both bounds of its syntenic search interval is unambiguous, and **None** if no edge in its syntenic search interval(s) is unambiguous. **Scrambled** means all syntenic search intervals for that gene have ambiguous edges and are between syntenic links (i.e. are Between). **Unassembled** means the gene may be in an unassembled region of the genome (one edge of the search interval is flush against a terminus of a scaffold).

	synder search interval classifications						
Species	Between	Lo	Hi	Both	None	Scrambled	Unassembled
A. lyrata	3048	32701	32789	17514	1550	1749	871
C. rubella	5139	32140	32021	18747	2116	2573	754
B. rapa	7404	22241	22331	6778	7810	6404	4788
E. salsugineum	7545	30161	30227	17304	3586	3976	306
S. paradoxus	699	5822	5814	5226	340	328	1038
S. mikatae	3266	3980	4003	3370	2003	2027	3027
S. kudriavzevii	4135	2628	2655	2022	3312	3411	4568
S. arboricola	4488	5137	5154	4856	1101	2149	513
S. eubayanus	4972	4729	4724	4462	1541	2883	481
S. uvarum	4987	2759	2754	2376	3430	3968	4256

`fagin` infers the search intervals by calling the `synder` search function. This is a function of a synteny map and four parameters: 1) `trans` specifies the function needed to transform the score column in the synteny map to one that is additive; 2) k is the number of conflicting syntenic intervals allowed in a block before it is broken (set to 0 by default); 3) r is a score decay rate that is used in calculating a score for each syntenic block created by `synder`; 4) `offsets` which specify the input and output bases (0 or 1) of the synteny map. Parameters 1 and 4 are specific to the tool that created the synteny map.

The output of the `synder search` function is: 1) a set of one or more search intervals for each focal gene (summarized in **Table S4**) and 2) a description of each search interval consisting of a flag describing each edge of the interval, whether the search interval overlaps a syntenic region in the synteny map, and a relative score for the search interval. The search intervals can be classified as shown in **Table S5**.

S3 `fagin` validation and parsing of GFF files

The GFF format is simple but highly error prone and GFFs from difference sources can follow very different conventions. This complicates practical analysis. While more standardized formats exist for storing feature information, GFF has persisted as the most commonly used. For this reason, `fagin` uses GFF, but also performs extensive validation and cleaning. The most problematic component of GFF is the 9th column that stores tag-value data. It is

from these tag-value pairs that we build the gene models that we use to extract the locations of features, the protein sequences for genes, and the RNA sequences of transcripts.

`fagin` perfoms the following checking and cleaning steps:

- Assert that all columns have correct type (see **Table S7**)
- Unify type synonyms (see **Table S6**)
- Handle AUGUSTUS fields. The AUGUSTUS gene prediction program uses the tag 'Other' to represent the Parent relationship. If the `source` column of the GFF3 file (2nd column) is "AUGUSTUS", then the tag 'Other' will be converted to 'Parent' (with a warning that will be passed through `rmonad` to the user).
- Assert that each `Parent`, `ID`, and `Name` tag contains a unique value. In the GFF3 spec, a tag can be associated with a comma-delimited list of values. `fagin` currently does not support this and will raise an error if this case is found.
- Treat `Parent` tags with a value of '-' as missing.
- Handle unnamed fields. If no `ID` is given, but there is one untagged field, and if there are no other fields, then cast the untagged field as an `ID`. This is needed to accommodate the irregular output of AUGUSTUS.
- Assert parent child relations are correct.
  - If a feature links to a `Parent`, then the `Parent` must exist in the GFF
  - All `Parent IDs` must have either type `gene` or `mRNA`
  - All `CDS` and all `exon` must have a `Parent` (`gene` or `mRNA`)
  - All `mRNA` and `IDs` must be unique

Table S6: GFF type (3rd column) equivalence groups. According to the GFF3 specification `https://github.com/The-Sequence-Ontology/Specifications`, names for the 3rd column of a GFF3 file should contain the names or IDs of elements from the Sequence Ontology [53]. Based on this, we coalesce members from the equivalence groups described in this table. The names listed in the **right** column (equivalent terms) are converted to the name in the **left** column. `SO:XXXXXXX` terms are Sequence Ontology IDs. We merge the `mRNA` and `transcript` groups; these groups are technically different, but they are often used interchangeably in practice. Also we have merged the `exon` and the more specific `coding_exon` terms.

term	equivalent term
gene	`SO:0000704`
mRNA	`messenger_RNA`, `messenger RNA`, `SO:0000234`, `transcript`, `SO:0000673`
CDS	`coding_sequence` , `coding sequence`, `SO:0000316`
exon	`SO:0000147`, `coding_exon`, `coding exon`, `SO:0000195`

Table S7: A GFF file is a tab-delimited file with optional comments ('#' initialized). The table must have nine columns with the types listed under column **Base Type**. Entries in some columns are optional ('.' indicates optional). The phase column is required to be given for all GFF. The "attr" column contains a semicolon delimited list of `tag=value` pairs. According to the GFF3 specification, values may be comma delimited lists, but `fagin` does not currently handle these lists and raises an error if a comma appears in one of the tags required by `fagin` (`Parent`, `ID`, or `Name`).

Column name	Base type	Optional	Notes and Restrictions
seqid	string	No	all IDs present in genome
source	string	Yes	
type	string	No	
start	integer	No	
end	integer	No	$end \geq start$
score	numeric	Yes	
strand	$+\|-$	Yes	
phase	0|1|2	Yes	Required for CDS features
attr	tag-value list	No	

S4 `fagin` data extraction from GFF and genome files

Get mRNAs. Given the GFF and genome, the extraction of the transcripts (mRNAs) is fairly straightforward. The sequences of all exons are extracted and concatenated. If the sense of the mRNA is negative, the result is then reverse transcribed.

Get proteins. Extracting the protein coding sequences from the genome given the GFF is slightly more involved. The GFF records all Coding Sequences (CDS) and associates each with a parent (an mRNA or gene feature). The CDS may be spread across many exons, and thus be a list of DNA intervals. These DNA intervals can be extracted from the genome and pasted together to form the full CDS. However, there is some nuance to this step. First, if the mRNA is negative sense, the CDS must be reverse transcribed. A more difficult case arises when the initial interval of the CDS does not begin in the correct reading frame. This can happen, for example, when part of the gene model is missing from the assembly. So the first interval in the CDS may begin on the 2nd or 3rd position on the codon. The 'phase' column of the GFF stores the number of nucleotides that must be subtracted from the beginning of a CDS interval to read the first complete codon. `fagin` stores the phase data and will trim all models that start in a non-zero phase. Thus, partial protein models are allowed.

Once the CDSs, or partial CDSs, have been extracted, they may then they can be translated. `fagin` uses the `translation` function from the `Biostrings` package of the Bioconductor project. By setting `if.fuzzyi.codon="solve"`, it perform a "fuzzy" translation where codons with ambiguous nucleotides (e.g. N for unknown base or Y for pyrimidine) will either be translated as X (if more than one amino acid matches the pattern) or a specific amino acid (if only one amino acid matches). The resulting proteins are given the name of their parent and stored for future use.

Get ORFs in mRNAs and the genome. `fagin` identifies ORFs in the mRNAs and across

the entire genome. ORF identification is limited to the mono-exonic case (i.e. splice sites are not searched for). This is often reasonable since young genes tend to have few or no introns (though there are notable exceptions to this trend [8]). `fagin` uses the Bioconductor ORFik package [54] to identify the longest, uninterrupted ORF for each stop codon in the genome (or mRNA). For the genome (but not the mRNA) it search both strands. The start and stop codons can be set by the user (the `fagin` default is `START=ATG` and `STOP=TAA,TGA,TAG`). The minimum ORF length can also be set by the user, with the default being 30 amino acids.

`fagin` currently use the standard gene table for all genes. This would cause problems in animal and fungi mitochondria and other cases where non-standard gene codes appear (plant organelle genomes use the standard gene code).

S5 `fagin` homology inference statistics

`fagin` calculates sequence similarity for proteins and DNA through Smith-Waterman alignments of the query features of the focal genome (e.g., the gene sequence, spliced mRNA sequence, or translated coding sequence) against the features on the target genome that overlap the search interval. These alignments provide a score, but no direct measure of statistical significance. To estimate the statistical significance of sequence matches, `fagin` infers p-values from an estimated false-positive distribution.

To simulate the false positive distribution, `fagin` starts with *reversed* query sequences. The sequence reversal erases the homology signal while preserving the sequence composition and site-dependencies (assuming site-dependencies are symmetric). For DNA, the order of nucleotides is reversed but not complemented. The idea of using reversed sequences as a control has been explored in the past [55, 42]. For proteins, the order of amino acids is reversed. Reversed proteins have been used as a control to test sequence masking algorithms [56]). There is no natural process that will reverse the order of codons in a coding sequence (since this would require many independent tri-nucleotide inversions), or to reverse the order of bases in a nucleotide sequence (inversions would also take the complement of the bases).

`fagin` next compares randomly selected query/target pairs of sequences. For protein searches, k query proteins are randomly sampled with replacement. For each sampled query protein, one target is randomly selected for each target that overlaps a search space on the target genome. For nucleotide searches, the k query genes are each searched against a random search interval. The alignments of the reversed query genes against the randomly chosen target sequences provides scores that approximate draws from the false positive distribution.

The raw Smith-Waterman scores need to be adjusted to account for search space size. The search space size is the product of the length of the focal sequence and the summed lengths of all target sequences. To account for search space size, `fagin` replaces the score with an adjusted score:

$$S_i' = S_i + b_0 + b_1 log(m_i \sum_{j=1}^{J} n_j) \quad (2)$$

where S'_i is the adjusted score, S_i is the original raw alignment score, m_i is the length of the focal amino acid sequence, n_j is the length jth of the target sequence, and J is the total number of target sequences that will be searched. b_0 and b_1 are the regression coefficients for the robust regression of the raw scores on the search $log(mn)$ where mn is the search space size. Robust linear regression was performed using the R function `L1fit` from the `L1Pack` package which implements the Barrodale-Roberts algorithm for L1 linear approximation [57].

Once adjusted scores are obtained against random search intervals, `fagin` fits the simulated highest scores for each query gene to a Gumbel distribution (a model of maximum values) using the R packages `fitdistrplus` [58] (using the maximum goodness-of-fit estimation with Cramer-von Mises distance), and calculates one-sided p-values for observed hits from this distribution. Finally, the p-value for each query is adjusted for the number of target sequences (across all species) that it is searched against (using the Holm method by default).

`fagin` uses the local Smith-Waterman algorithm, as implemented in the Bioconductor Biostrings package [59], to align each query gene to each of the similar sequences found in its target-genome search interval(s). For protein sequences, `fagin` uses the BLOSUM80 substitution matrix by default (the user may choose a different one). Nucleotide sequences are aligned with a local Smith-Waterman algorithm with a gap opening penalty of 10 and gap extension penalty of 4.
